# Polyelectrolyte-Coated Mesoporous Bioactive Glasses via Layer-by-Layer Deposition for Sustained Co-Delivery of Therapeutic Ions and Drugs

**DOI:** 10.3390/pharmaceutics13111952

**Published:** 2021-11-17

**Authors:** Carlotta Pontremoli, Mattia Pagani, Lorenza Maddalena, Federico Carosio, Chiara Vitale-Brovarone, Sonia Fiorilli

**Affiliations:** 1 Department of Applied Science and Technology, Politecnico di Torino, Corso Duca degli Abruzzi 24, 10129 Torino, Italy; carlotta.pontremoli@unito.it (C.P.); mattia.pagani@polito.it (M.P.); chiara.vitale@polito.it (C.V.-B.); 2 Department of Chemistry, NIS Interdepartmental and INSTM Reference Centre, University of Torino, via Giuria 7, 10125 Torino, Italy; 3 Department of Applied Science and Technology, Politecnico di Torino, Alessandria Campus, Viale Teresa Michel 5, 15121 Alessandria, Italy; lorenza.maddalena@polito.it (L.M.); federico.carosio@polito.it (F.C.)

**Keywords:** bone regeneration, layer by layer deposition, drug delivery system, sustainable co-release, ibuprofen, copper ions

## Abstract

In the field of bone regeneration, considerable attention has been addressed towards the use of mesoporous bioactive glasses (MBGs), as multifunctional therapeutic platforms for advanced medical devices. In fact, their extremely high exposed surface area and pore volume allow to load and the release of several drugs, while their framework can be enriched with specific therapeutic ions allowing to boost the tissue regeneration. However, due to the open and easily accessible mesopore structure of MBG, the release of the incorporated therapeutic molecules shows an initial burst effect leading to unsuitable release kinetics. Hence, a still open challenge in the design of drug delivery systems based on MBGs is the control of their release behavior. In this work, Layer-by-layer (LbL) deposition of polyelectrolyte multi-layers was exploited as a powerful and versatile technique for coating the surface of Cu-substituted MBG nanoparticles with innovative multifunctional drug delivery systems for co-releasing of therapeutic copper ions (exerting pro-angiogenic and anti-bacterial effects) and an anti-inflammatory drug (ibuprofen). Two different routes were investigated: in the first strategy, chitosan and alginate were assembled by forming the multi-layered surface, and, successively, ibuprofen was loaded by incipient wetness impregnation, while in the second approach, alginate was replaced by ibuprofen, introduced as polyelectrolyte layer. Zeta-potential, TGA and FT-IR spectroscopy were measured after the addition of each polyelectrolyte layer, confirming the occurrence of the stepwise deposition. In addition, the in vitro bioactivity and the ability to modulate the release of the cargo were evaluated. The polyelectrolyte coated-MBGs were proved to retain the peculiar ability to induce hydroxyapatite formation after 7 days of soaking in Simulated Body Fluid. Both copper ions and ibuprofen were co-released over time, showing a sustained release profile up to 14 days and 24 h, respectively, with a significantly lower burst release compared to the bare MBG particles.

## 1. Introduction

Since Vallet Regì’s [1] and Zhao’s [2] groups proposed for the first time the use of mesoporous bioactive glasses (MBGs) for biomedical applications, their role in the bone regeneration field [3] and soft tissue applications [4] were extensively studied. To date, MBGs continued to receive considerable attention as multifunctional biomedical devices [5], due to the possibility to combine their capability to induce hydroxyapatite deposition with the ability to store and release therapeutic species exploiting their mesoporous structure. In fact, their extremely high exposed surface area and pore volume allow the loading of active agents, such as drugs or growth factors, and their chemical composition can be easily tailored for specific applications [6,7] through the incorporation of selected metal ions (i.e., Cu, Sr) during the synthesis. By following this strategy, a single biomaterial can be designed and enriched with several therapeutic abilities, spanning from pro-osteogenic [8,9] to pro-angiogenic or anti-bacterial properties [10].

Based on these considerations, MBGs could play a pivotal role in the field of bone healing application as controlled drug delivery systems and can be considered excellent candidates as multifunctional delivery platforms for prolonged and localized release of therapeutic agents (ions, drugs, growth factors), in order to simultaneously target the several causes connected to an impaired bone healing. In fact, conventional administration routes, such as oral administration and injection, resulting in low levels of the therapeutic agents and often present various side effects. The low levels of therapeutic agents in the tissue are due to poor blood circulation and inadequate tissue penetration characteristics of bone tissue. Therefore, an efficient and adequate delivery in the pathological site is not achieved [11]. At variance, sustained drug release from biomaterials could enhance the delivery efficiency, maintaining a suitable therapeutic dose over time directly at the pathological site, reducing, in the meantime, the related side effects.

Over the last years, the scientific community devoted great efforts to develop drug delivery systems by combining active agents with biocompatible materials in order to achieve a sustained local release [12,13]. MBGs, due to their excellent bioactivity and peculiar structural features, represent one of the most promising biomaterials, perfectly owning all the requirements to conceive advanced devices for drug delivery in the field of bone tissue repair [14]. To date, despite their excellent drug loading capacities, some drawbacks are still limiting MBG clinical translation, in particular, the strong burst release of incorporated drugs once in contact with body fluids due to the open mesopore structure.

Many attempts have been committed to designing formulations based on MBGs able to modulate the cargo release kinetics, such as a surface modification or the combination with polymeric vehicle phase to produce a hybrid formulation [15,16,17]. By exploiting the high number of terminal hydroxyl groups, the MBG surface can be easily functionalized, achieving several targets such as the reduction of particle aggregation and nonspecific surface adhesion [18,19,20] or an improvement of drug loading capacity and longer drug release time [1,21,22,23,24].

Despite the efficacy of these promising functionalization approaches, most of these explored strategies are based on covalent binding and usually require several synthetic steps involving potentially toxic by-products, which can lead to an enhancement of the material’s cytotoxicity. As an alternative, the layer-by-layer (LbL) deposition can be considered a faster and greener approach, offering a promising possibility for the noncovalent engineering of surfaces to modulate the release behavior of therapeutic agents [25]. More in detail, the LbL deposition involves the electrostatic interactions between oppositely charged polyelectrolytes, which are alternatively assembled on the outer surface [26]. Usually, the layer deposition is repeated several times, leading to the formation of thermally and mechanically stable polyelectrolyte multi-layers (PEMs) [27]. The control of the deposition parameters, such as the ionic strength, the pH, or the temperature, allows obtaining PEMs with peculiar features in terms of layer organization, thickness, surface charge, and wettability [28]. In addition, the wide range of materials, which could be coated, both as flat substrates or in the form of particulate (including glass, quartz, silicon wafers, mica, and different polymers) fostered the research interest in this field, highlighting the potential of LbL technology for various applications, spanning from fuel-cells and dye-sensitized solar cells preparation, biosensor and gas sensor preparation to thin films deposition for anti-reflection or flame retardant treatment [29,30,31]. Depending on the final application, the multi-layers can be tailored by using synthetic or natural polymers, or their mixture [32]. Among the different classes of polyelectrolytes, in the field of biomedical applications, it is essential to privilege the use of natural polyelectrolytes, such as components of the extracellular matrix (hyaluronate, collagen, elastin, fibronectin, laminin), proteins (protamine, gelatin), nucleic acids (DNA and RNA) and biopolymers (alginate, chitosan, silk fibroin) [33,34], in order to avoid any cytotoxic effect induced by the coating and maintain the overall biocompatibility of the material.

Since PEMs can also be assembled on the surface of nano- and micro-particles, the LbL deposition could be considered as a promising strategy to design drug delivery systems to control the payload release profile, in which the therapeutic agents could be both incorporated into the multi-layered structures or, the alternative, themselves act as charged polyelectrolyte. Several examples of LbL applications in the field of drug delivery systems are reported in the literature, both by exploiting the polyelectrolyte multi-layers to design microcapsules and to develop a multifunctional coating. Shi and Caruso [35] investigated the release profiles and rates of pyrene from microcapsules composed of PEMs, reporting that the release rate was tailored by modifying the number of polyelectrolyte layers. Yang et al. [36] produced mesoporous silica nanoparticles loaded with antitumor drug and then coated with alginate and chitosan, which are well known as biodegradable and biocompatible polymers by using the LbL technique. The developed system was able to act as pH-sensitive gatekeepers, enabling the release of drug molecules once in contact with an acidic environment. Moreover, the authors performed cytotoxicity assays and reported higher biocompatibility for the polymer-coated compared to the bare samples. Zhou et al. [26] employed alginate and chitosan to coat poly (lactide-*co*-glycolide) (PLGA) nanoparticles to develop a biocompatible drug delivery system able to impart antifouling properties in addition to reduce nonspecific cell uptake. The authors demonstrated very low interaction with albumin, confirming the antifouling properties and a related decrease in the cellular uptake compared to the bare samples.

In this work, the LbL approach was successfully applied to the surface of MBG-based nanoparticles with the aim to design and characterize multifunctional biomaterials for tissue regeneration applications, to be used as drug delivery systems able to act as nanocarriers showing a sustained co-release of both therapeutic ions and anti-inflammatory drug. More in detail, the co-release of ibuprofen and copper ions was considered as a promising strategy for promoting healing of soft and hard tissues since the release of ibuprofen was expected to contrast the spreading of the inflammatory phase during the first stage of the healing process and, simultaneously, the release of copper ions was expected to stimulate the angiogenesis processes and exert antimicrobial activity, overall promoting regeneration. Recently in this regard, the dual therapeutic effect of copper ions released by MBG nanoparticles was finely proved by Paterson et al. [37], who showed remarkable anti-bacterial activity against both planktonic and biofilm bacteria with a broad spectrum of antimicrobial action, whilst showing no cytotoxicity in either 2D cell monolayers or a 3D human skin model. In addition, a clear proangiogenic effect of both MBGs and Cu-containing MBGs was also confirmed by an increase in endothelial cell outgrowth seen at concentrations between 30 and 300 μg/mL.

The authors recently reported the development of injectable hybrid formulations for bone healing applications by combining poly (ether urethane) (PEU) hydrogels with Cu-containing MBGs loaded with ibuprofen with the final goal to simultaneously promote angiogenesis, anti-microbial, and anti-inflammatory effects [16] and proved the ability of the resulting system to modulate the ion/drug co-release, avoiding the undesired burst effect, typical of MBGs. Inspired by these promising results, in this contribution, by simply exploiting the negatively charged surface of the MBG, we investigated the LbL deposition of polyelectrolytes on the surface of Cu-substituted MBG nanoparticles loaded with ibuprofen as an alternative strategy to achieve a sustained co-release of copper ions and drug.

In particular, thanks to the possibility to load the ibuprofen both in the mesopores of the MBG and as PEM in the multi-layered coating, two different LbL assembly routes were investigated, and the release properties of the resulting systems were compared. In the first strategy, the polyelectrolytes chitosan and alginate were selected as natural biodegradable and biocompatible polymers and were alternatively deposited by exploiting their opposite positive and negative charges, respectively. Once obtained the LbL coated-MBGs, ibuprofen was loaded into the MBG pores by using the incipient wetness technique. In the second approach, the alginate layer was replaced by the ibuprofen (loaded as polyelectrolyte layer) by exploiting the ibuprofen negative charge (COO^−^), able to electrostatically interact with the chitosan positive charges. Since the LbL deposition is usually conducted on flat surfaces, the optimization of the overall procedure to coat the MBG particle surface required different adjustments in terms of the number of deposited layers in order to preserve the peculiar structural and chemical features of the MBGs (i.e., morphology, ion release and bioactivity). The two obtained systems were then evaluated in terms of capability to avoid the burst release observed in the uncoated samples and to modulate the release rate of both the incorporated copper ions and loaded ibuprofen.

## 2. Materials and Methods

### 2.1. Materials

Cetyltrimethylammonium bromide (CTAB ≥ 98%), NH_4_OH (Ammonium hydroxide solution), Tetraethyl orthosilicate (TEOS, Tetraethyl orthosilicate, reagent grade 98%), Ca (NO_3_)_2_·4H_2_O, 99%), Copper chloride (CuCl_2_ 99%), ibuprofen (>98% GC), Chitosan (low molecular weight), Alginic acid (sodium salt), Sodium hydroxide, Hydrochloric acid (ACS reagent, 37%), and Trizma^®^ base, Primary Standard and Buffer, ≥99.9% (titration) were all purchased from Sigma Aldrich, Milan, Italy and used as received without any purification.

### 2.2. LbL-Coated Cu-Substituted MBGs as Ibuprofen Delivery System

**Figure 1:**
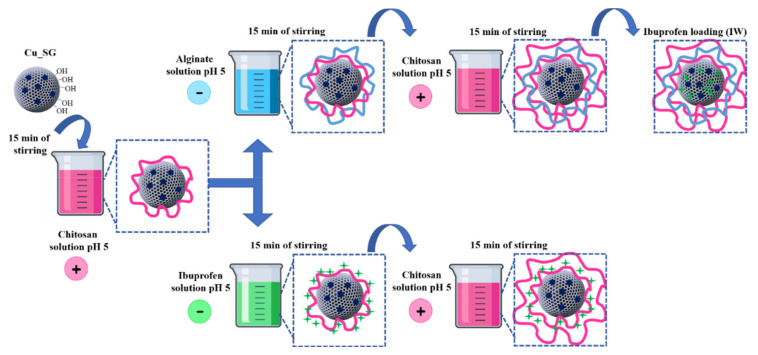
Schematic illustration of the two different routes adopted to produce drug delivery systems able to differently modulate the release kinetics of both copper ions and ibuprofen.

Cu-substituted MBGs nanoparticles (nominal molar ratio Cu/Ca/Si = 2/13/85, hereafter named as Cu_SG) were prepared using a base-catalyzed template sol-gel synthesis, following the protocol optimized by the authors [15,16]. The multi-layered surface was obtained by following two different routes, as reported schematically in Figure 1, in order to produce drug delivery systems able to differently modulate the release kinetics of both copper ions and ibuprofen. In the first strategy, chitosan and alginate were assembled by forming the multi-layered surface, and, successively, ibuprofen was loaded by incipient wetness impregnation, following the protocol optimized by the authors [16]. On the contrary, in the second strategy, alginate was replaced by ibuprofen by exploiting the ibuprofen negative charge of deprotonated carboxylic moieties (COO^−^), able to electrostatically interact with the chitosan positive charged groups.

#### 2.2.1. Cu-Substituted MBG Nanoparticles

6.6 g of CTAB, acting as template agent and 12 mL of NH_4_OH were dissolved in 600 mL of ddH_2_O under magnetic stirring (350 rpm) for 30 min. Then, 30 mL of TEOS, 4.888 g of Ca(NO_3_)_2_ ∙ 4H_2_O and 0.428 g of CuCl_2_ were added, and the obtained suspension was vigorously stirred for 3 h at room temperature (RT). The powder was collected by centrifugation (Hermle Labortechnik Z326, Hermle LaborTechnik GmbH, Wehingen, Germany) at 10,000× *g* rpm for 5 min, washed twice with ddH_2_O, and once with absolute ethanol. The collected powder was dried at 70 °C for 12 h and calcined at 600 °C in air for 5 h at a heating rate of 1 °C min^−1^ using a Carbolite 1300 CWF 15/5 (Carbolite Ltd., Hope Valley, UK), in order to remove CTAB completely.

#### 2.2.2. LbL Deposition of Chitosan and Alginate: First Strategy

Chitosan (Chi) and alginate (Alg) were assembled in a concentration of 1 mg/mL in water by following the procedure reported in the literature [26]. The pH value of each electrolyte solution was adjusted to 5 by the addition of glacial acetic acid or 1 M NaOH. For the assembly of Chi/Alg multi-layers on the MBG surface, the deposition time of each polyelectrolyte layer was 15 min. After each polyelectrolyte deposition, particles were centrifuged at 10,000× *g* rpm for 3 min and washed 3 times with water before deposition of the next layer. Initially, in the preliminary investigation, up to 10 layers were deposited on the MBG surface. However, based on FE-SEM observations, the samples coated with more than 4 layers showed the undesired formation of particle agglomerates embedded in a polymer phase. On the contrary, the deposition of 3 layers was found to be optimal to obtain multi-layered coated particles, avoiding the formation of agglomerates and preserving the peculiar features of the MBGs (morphology, size, bioactive behavior). Based on these considerations, 3 layers were deposited, a first layer of chitosan, a second of alginate, and the last one of chitosan, and the related material was referred to as Cu_SG_CAC.

Ibuprofen was loaded into Cu_SG_CAC through the incipient wetness method [16,38]. In brief, 0.1 g of Cu_SG_CAC were impregnated several times by dropping consecutive small aliquots of an ibuprofen solution in ethanol (at the final concentration of 30 mg/mL) onto the powders at RT. After each impregnation, ethanol was evaporated at 50 °C for 10 min, and the dried powder was mixed with a spatula. In order to completely fill the mesopores with ibuprofen, the impregnation procedure was carried out with 4 × 100 μL aliquots. Lastly, the obtained powders were dried at 50 °C overnight and named as follows: Cu_SG_CAC_Ibu.

#### 2.2.3. LbL Deposition of Chitosan and Ibuprofen: Second Strategy

Chitosan and ibuprofen were assembled in a concentration of 1 mg/mL in water and ethanol, respectively. The pH value of each electrolyte solution was adjusted to 5 by the addition 1 M NaOH. For the assembly of the multi-layers on the MBGs surface, the deposition time of each polyelectrolyte layer was 15 min. After each polyelectrolyte deposition, particles were centrifuged at 10,000× *g* rpm for 3 min and washed 3 times with water and once with ethanol before deposition of the next layer. Three layers were deposited, the first layer of chitosan, a second of ibuprofen, and the last one of chitosan, and the related material were referred to as Cu_SG_CIC.

### 2.3. Physico-Chemical Characterization of LbL-Coated Cu-MBGs

The morphology of bare and coated MBG nanoparticles was analyzed by Field-Emission Scanning Electron Microscopy (FE-SEM) using a ZEISS MERLIN instrument (Oberkochen, Germany). For FE-SEM observations, 10 mg of the samples were dispersed in 10 mL of isopropanol using an ultrasonic bath (Digitec DT 103H, Bandelin, Berlin, Germany) for 5 min to obtain a stable suspension. The resulting suspension was dropped onto a copper grid (3.05 mm Diam.200 MESH, TAAB, Aldermaston, Berks, UK), dried and successively chromium-coated prior to imaging (Cr layer of ~7 nm).

Transmission Electron Microscopy (TEM) imaging was performed on a JEOL JEM300FEG electron microscope equipped with an ISIS 300 X-ray microanalysis system (Oxford Instruments, Abingdon-on-Thames, UK). The uncoated Cu_SG sample was ultrasonically dispersed in n-butanol and transferred to carbon-coated nickel grids prior to the image acquisition.

Textural properties were analyzed by N_2_ adsorption-desorption measurement conducted by ASAP2020, Micromeritics analyzer (ASAP 2020 Plus Physisorption, Norcross, GA, USA) at a temperature of –196 °C, and before measurements, samples were outgassed at 150 °C for 5 h. The Brunauer–Emmett–Teller (BET) model equation was used to calculate the specific surface area (SSA_BET_) from the adsorption branch of the isotherm in the 0.04–0.2 relative pressure range. The mesoporous silica pore size distribution was calculated through the DFT method (Density Functional Theory) using the NLDFT kernel of equilibrium isotherms (desorption branch).

The multistep deposition of selected components was characterized by Fourier Transform infrared spectroscopy (FT-IR), thermo-gravimetric analysis (TGA), and ζ potential measurements. FT-IR spectra were collected using an FT-IR spectrometer (Bruker Equinox 55 spectrometer) in the 4000–400 cm^−1^ wavenumber range. TGA was conducted on a TG 209 F1 Libra instrument (Netzsch, Erich NETZSCH GmbH & Co. Holding KG, Selb, Germany) over a temperature range of 25–600 °C with a heating rate of 10 °C/min under air in a flow of 50 mL/min). ζ-potential measurements (Zetasizer nano ZS90 Malvern Instruments Ltd., Malvern, UK) were conducted in aqueous media, and 3 analyzes repeated for each sample (shown data are the means ± standard deviations).

### 2.4. Copper Ions and Ibuprofen Release from LbL-Coated MBGs

Release studies of both ibuprofen and Cu^2+^ ions were conducted at 37 °C using Trizma^®^ as a release medium by using nanoparticle suspension with a concentration of 20 mg/mL. In particular, 40 mg of powder were suspended in 2 mL of buffer up to 24 h at 37 °C in an orbital shaker (Excella E24, Eppendorf, Germany) with an agitation rate of 150 rpm. At predefined time points (1 h, 3 h, 5 h, 8 h, 24 h), the suspension was centrifuged at 10,000× *g* rpm for 5 min by using a Hermle Labortechnik Z326 (Hermle LaborTechnik GmbH, Wehingen, Germany), the supernatant was collected and replaced by the same volume of fresh buffer solution to keep constant the volume of the release medium. The release experiments were carried out in triplicate. The concentration of the ibuprofen was measured by UV-Vis spectrometer by measuring the absorbance at 274 nm. The collected extracts were also analyzed by ICP to measure the concentration of released copper ions. In order to express the results in terms of released percentage and to evaluate the total amount of ions incorporated during the synthesis, the powders were dissolved in a mixture of nitric and hydrofluoric acids (0.5 mL of HNO_3_ and 2 mL of HF for 10 mg of powder), and the resulting solutions were analyzed via ICP analysis. Each experiment was performed in triplicate and data were presented as means ± standard deviations.

Since the copper release did not reach the plateau at 24 h, to properly evaluate the copper release profile and assess the effect on the release kinetics due to the presence of the multi-layer deposition, the copper release test was prolonged up to 14 days by adding further time points (2 d, 3 d, 8 d, 10 d, 14 d).

### 2.5. In Vitro Bioactivity of LbL-Coated MBGs

Since the bioactivity of the MBGs was considered an essential feature for promoting bone regeneration and thus one of the fundamental properties of the developed materials, bioactivity test was carried out on LbL coated MBGs by following the protocol reported by Maçon et al. [39] in which the authors described a unified method to evaluate the apatite-forming ability of the bioactive glasses.

In detail, the in vitro bioactivity test was performed by soaking the particles in Simulated Body Fluid (SBF) for up to 14 days. In brief, 30 mg of Cu_SG_CAC_Ibu and Cu_SG_CIC were soaked in 30 mL of SBF (final concentration 1 mg/mL), following the protocol described in the literature [39] at 37 °C up to 14 days in an orbital shaker (Excella E24, Eppendorf) with an agitation rate of 150 rpm. At each time point (3 h, 1 day, 3 days, 7 days and 14 days), the suspension was centrifuged at 10,000× *g* rpm for 5 min, the collected powder was washed twice with distilled water and dried in the oven at 70 °C for 12 h prior FE-SEM and XRD analysis to evaluate the apatite layer formation. Moreover, the pH of each recovered supernatant was measured to assess if the values were suitable for allowing osteoblasts to maintain their physiological activity [40].

## 3. Results and Discussion

### 3.1. Morphological, Structural, and Chemical Characterization of Cu_SG_CAC_Ibu

**Figure 2:**
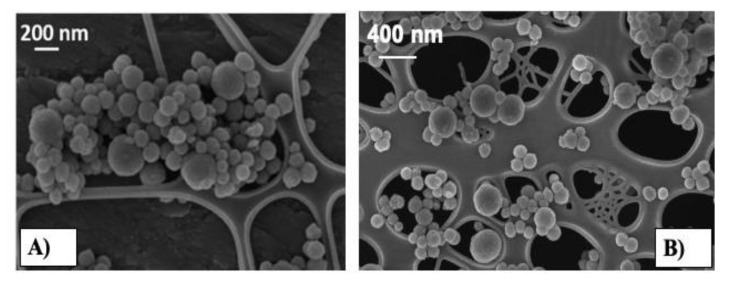
FE-SEM images of (**A**) Cu_SG_CAC_Ibu and (**B**) Cu_SG_CIC.

FE-SEM images of Cu_SG_CAC_Ibu, reported in Figure 2A showed nanoparticles with a size ranging between 100 and 300 nm. Compared to the bare samples, in which the homogeneous average size was reported to be around 150 nm (also confirmed by the TEM image, as reported in Figure S1) [15,16], a slight increase of about 50–100 nm, as well as heterogeneity in particle size, were observed, ascribed to the occurrence of the surface modification upon the deposition of the chitosan and alginate layers. Based on ellipsometric measurements reported in the literature, the layer thickness when chitosan and alginate were deposited on a flat silicon wafer was expected to increase linearly with the number of layers of about 10 nm [25,41,42]. Hence, assuming the deposition of three layers on a spherical surface, the overall diameter increase associated with the CAC layer deposition was expected to be around 60 nm, finely aligned to the increase in size observed for the Cu_SG_CAC_Ibu compared to the bare particles. Despite this assumption, the assessment of the homogeneous coating on the spherical surface of the particles and the coating thickness cannot be clearly evaluated by using FE-SEM images. In fact, the thickness of the multi-layer system is usually evaluated by ellipsometric measurements or Atomic Force Microscopy (AFM) after the deposition on a flat substrate, which allows the homogeneous coating of the surface [43]. However, FE-SEM observations confirmed that the LbL procedure and the subsequent ibuprofen loading did not significantly affect the morphology of both the particles.

Figure 2B shows the FE-SEM observations of Cu_SG_CIC. Concerning the Cu_SG_CIC, as already notified for the Cu_SG particle coated with CAC, nanoparticles with a size ranging between 100 and 300 nm were obtained, showing the same morphology of the corresponding bare samples Cu_SG. In some isolated cases, the build-up of multi-layers on the particles surface was accompanied by a slight increase in particle size, as observed for the Cu_SG_CAC_Ibu sample [25]. Since the multi-layer deposition with chitosan and ibuprofen has never been reported in the literature and the growth in the size of chitosan and ibuprofen layers cannot be compared with similar works, the layer thickness of the CIC samples cannot be clearly estimated by FE-SEM observations, even if a lower increase can be predictable due to the smaller steric hindrance of ibuprofen molecule compared to the alginate chain. Based on the morphological evaluation, it can be concluded that irrespective of the adopted LbL deposition procedure, the overall morphology of the coated MBG nanoparticles resulted mostly unaffected.

Since the LbL deposition could induce an undesired release of copper ions during the procedure, the chemical composition of coated copper-substituted MBGs was evaluated via ICP analysis after the dissolution of the particles in a mixture of nitric and hydrofluoric acids, evidencing that the amount of copper content resulted unaffected, confirming that the LbL deposition did not induce any loss of initial copper amount.

**Figure 3:**
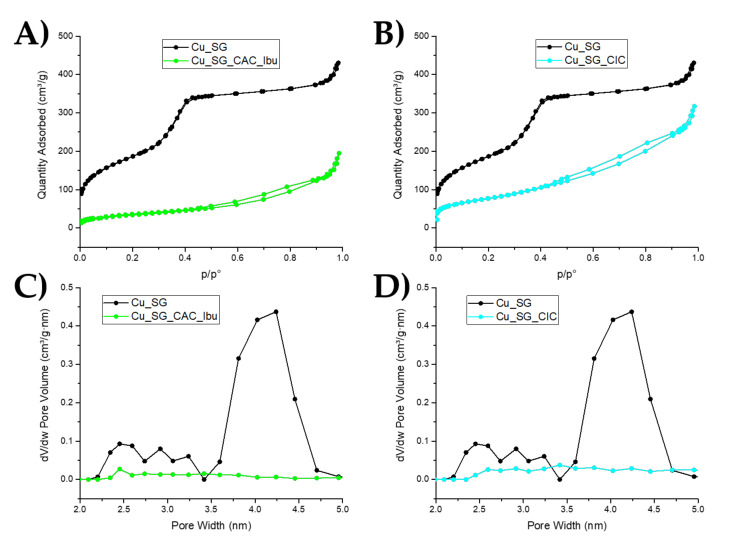
Type IV isotherm of (**A**) Cu_SG_CAC_Ibu and (**B**) Cu_SG_CIC and pore size distribution of (**C**) Cu_SG_CAC_Ibu and (**D**) Cu_SG_CIC.

N_2_ adsorption-desorption was carried out both on bare and analog coated samples to analyze the specific surface area, pore size, and pore volume. Both Cu_SG_CAC_Ibu and Cu_SG_CIC showed type IV isotherms, substantially different compared to that of the analog sample before LbL deposition (Figure 3A,B). As expected, the amount of adsorbed nitrogen and the calculated specific surface area was significantly lower in the case of the LbL coated samples. The almost full coverage of the mesopores by the deposited layers is shown in Figure 3C,D, where a drastic pore volume reduction was reported, at variance with the uncoated sample, showing average pore size of 4 nm. As reported in Table 1 and Figure 3 Cu_SG_CAC_Ibu showed a more extensive decrease of the specific surface area and pore volume, suggesting that the deposited layers fully blocked the entrance of mesopores. Cu_SG_CIC showed a smaller decrease in specific surface area and pore volume compared to Cu_SG_CAC_Ibu. These results suggest that ibuprofen loaded through the incipient wetness method was mostly adsorbed deep inside the mesopores, contributing to a further decrease of surface area and pore volume compared to the Cu_SG_CIC, where the drug molecules were mostly entrapped into the multi-layers deposited at the external surface.

**Figure 4:**
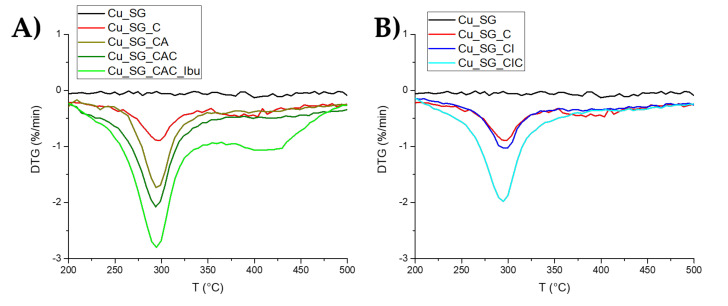
TGA first derivative for each loading step of (**A**) Cu_SG_CAC_Ibu and (**B**) Cu_SG_CIC, in the temperature range considered for the weight loss calculation.

In order to evaluate the weight increase associated with the deposited layer and ibuprofen loading, TGA analysis was performed after each layer deposition step and drug incorporation. Related results are reported in Figure 4, where, as expected, the peaks of the derivative curves showed larger weight loss after each layer deposition. Interestingly, in the first derivative of Cu_SG_CAC_Ibu, the curve shows a second broad peak in the range from 350 to 500 °C, ascribable to the loss of ibuprofen molecules loaded into the mesopores and/or partly entrapped in the multi-layers at the surface during the incipient wetness method. The calculations related to the range between 220 °C and 500 °C and reported in Table 2 indicate that the weight loss percentage associated with the first layer of chitosan was about 6.6% of the initial sample mass, while regarding the successive layers, the weight loss of the second alginate layer and the third chitosan layer was about 7.5% and 5.8%, respectively. The ibuprofen loading by incipient wetness method into CAC-coated sample resulted very efficient in terms of drug incorporation since the weight loss after this step results in 10.5% compared with the sample before drug loading.

The results of the TGA analysis of Cu_SG_CIC are reported in Figure 4B. The weight loss percentage after ibuprofen loading resulted very low, ~ 0.2%, suggesting a reduced drug loading capacity. Finally, the mass loss percentage of the third chitosan layer resulted similar to that reported for the first one.

**Figure 5:**
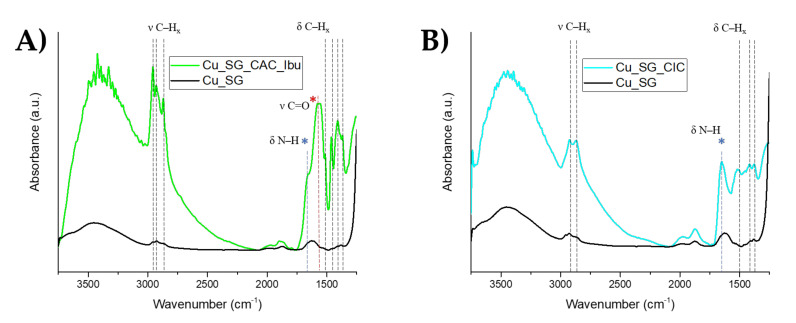
FT-IR spectra of (**A**) Cu_SG_CAC_Ibu and (**B**) Cu_SG_CIC. Black lines denote the C–H stretching and bending, and the red line and asterisk indicates the C=O stretching of the carboxylate groups and the blue line and asterisk indicate the N–H bending.

FT-IR spectra of LbL coated samples allowed to detect the presence of the peaks associated with chitosan, alginate, and ibuprofen. Figure 5 reports the spectra of Cu_SG_CAC_Ibu and Cu_SG_CIC compared with the bare sample.

The large absorption detectable for both spectra at around 3500 cm^−1^ was ascribed to stretching vibration of the NH_2_ and OH groups engaged in H-bonding [44,45,46]. The peaks in the range of 2871–2959 cm^−1^ can be attributed to CH symmetric and asymmetric stretching of chitosan and alginate for Cu_SG_CAC_Ibu and of chitosan for Cu_SG_CIC typical of polysaccharide molecules [46]. The shoulder at around at 1650 cm^−1^ present in both Cu_SG_CAC_Ibu and Cu_SG_CIC spectra (blue asterisk) can be ascribed to the N–H bending of the pronated amino group of chitosan.

The intense peak at around 1560 cm^−1^ (red asterisk) only observable for Cu_SG_CAC_Ibu sample is ascribable to C=O stretching of the carboxylate groups (COO^−^) of the alginate layer [44,46] and ibuprofen molecule. At variance, this band is clearly not detectably visible for the Cu_SG_CIC spectrum, most likely due to the low quantity of loaded ibuprofen. In the last region of the spectra (1369–1458) the peaks can be imputed to CH_2_ bending of the chitosan, alginate, and ibuprofen [41,43].

**Figure 6:**
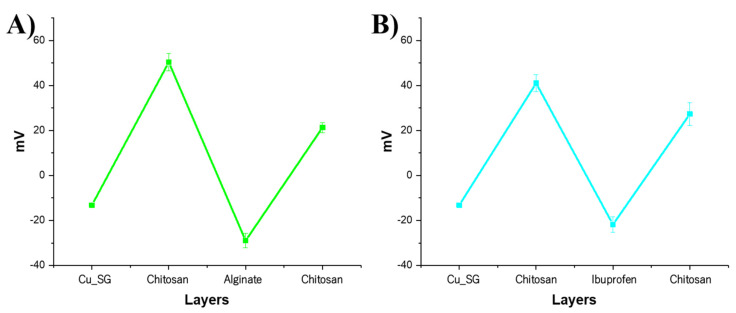
Potential measurement of (**A**) Cu_SG_CAC_Ibu, and (**B**) Cu_SG_CIC after each layer deposition.

In order to evaluate the correct alternative deposition of chitosan, alginate, and ibuprofen, the ζ-potential was measured after the addition of each polyelectrolyte layer. Along with confirming the presence and coverage of the polymer coating, ζ-potential measurements allowed to assess the stability of particle suspensions. In fact, ζ-potential values above +20 mV or around −20 mV can usually be considered an indication of stability and enhanced uniformity, ascribed to the strong repulsion forces among particles, allowing to prevent aggregation [47]. As shown in Figure 6 and reported in Table 3, for all samples, after the first deposition of chitosan, the surface charge moved from negative values (due to the deprotonation of surface silanols) to positive values due to the positively charged amine groups of the chitosan. The deposition of the second layer of alginate or ibuprofen induces a charge inversion on the surface due to the negatively charged carboxylate groups of alginate and ibuprofen, followed by the last charge inversion after the deposition of the third layer of chitosan. The last layer of chitosan showed a positive charge, confirming the occurrence of the stepwise deposition driven by the electrostatic interactions. In addition, the high values measured for all the analyzed samples indicate the high stability of the particle suspension.

### 3.2. Bioactive Behavior of LbL-Coated MBGs in SBF

**Figure 7:**
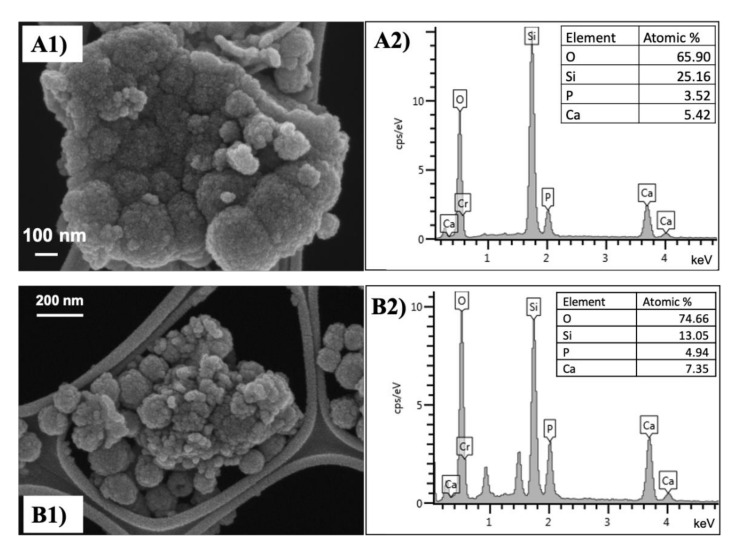
FE-SEM observation (**A1**) and EDS spectrum (**A2**) of Cu_SG_CAC_Ibu and FE-SEM observation (**B1**) and EDS spectrum (**B2**) of Cu_SG_CIC after 7 days of soaking in SBF.

FE-SEM images of Cu_SG_CAC after soaking in SBF clearly confirmed that the layer-by-layer deposition and the successive ibuprofen loading did not hinder the bioactive behavior of Copper-substituted MBG samples. Compared to the corresponding bare samples, a slowing delay in the deposition of the hydroxyapatite layer was observed for both samples. In fact, a rough layer of globular agglomerates on the surface of the particles appeared after 7 days of soaking (Figure 7(A1)) and not after just 1 day, as observed for the corresponding bare samples. As shown in Figure 7(A2), the presence of HA crystals was revealed by the EDS analysis performed on powders, which evidenced a Ca/P ratio close to 1.7, typical of HA [48,49].

The slowdown in the HA deposition is ascribed to the presence of the polyelectrolyte multi-layered coating. In fact, the inter-diffusion of the shorter polymer chains of alginate into the longer polymer chains of chitosan, due to the possibility of molecular rearrangements subsequent to first contact [50], leads to a formation of a denser network [25,50]. This dense polymer network is expected to hinder to some extent, the ionic exchange reactions involved at the interface of the MBGs and the medium and is essential to start the HA formation.

Despite this delay, the Cu_SG_CAC_Ibu retained their ability to promote the HA formation, an essential feature for promoting bone regeneration.

As far as the Cu_SG_CIC is concerned, FE-SEM images evidenced that hydroxyapatite formation occurred after only 1 day of soaking, resulting in a compact layer of needle-such as nanocrystals covering the particle surface. The agglomerates of apatite-like phase increased in size during the test, causing the full embedding of the particle surface after 7 days of soaking, as highlighted in Figure 7(B1). EDS analysis, reported in Figure 7(B2), further confirms the presence of HA, revealed by the appearance of phosphorous and a Ca/P ratio very close to 1.7, the typical value reported in the literature for hydroxyapatite. Unlike the layer-by-layer deposition strategy reported previously (Cu_SG_CAC_Ibu sample), the polyelectrolyte multi-layered coating based on chitosan, ibuprofen, and chitosan seems to not delay the deposition of the hydroxyapatite layer, which results unaffected compared to the corresponding bare samples [15,16]. This behavior could be ascribed to the low molecular weight of the ibuprofen, that compared to the steric hindrance of alginate, lead to a formation of a more open network, and could be explained from different modes of reaction between ibuprofen/chitosan and alginate/chitosan. In fact, some extents of ibuprofen could stick to chitosan on first contact (diffusion-limited mode of interaction), limiting the molecular rearrangement [50], and thus leading to a more open network, which allows the faster ion exchanges essential for the HA formation. On the contrary, the diffusion of alginate into the longer polymer chains of chitosan allows molecular rearrangements subsequent to first contact, forming a denser network [25], responsible of the delay in the ionic exchange reaction.

Both Cu_SG_CAC_Ibu and Cu_SG_CIC retained their ability to induce HA formation, allowing to preserve an essential feature of this material for application in bone regeneration processes.

### 3.3. Copper Release from Cu_SG_CAC_Ibu and Cu_SD_CAC_Ibu

**Figure 8:**
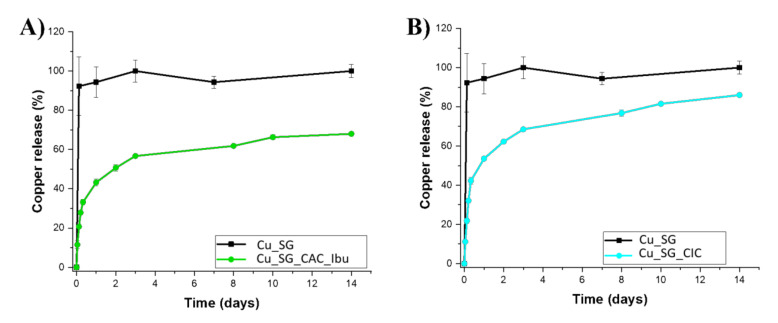
Copper release profile of (**A**) Cu_SG_CAC_Ibu and (**B**) Cu_SG_CIC in Tris-HCl compared to the bare sample Cu_SG.

The copper release profile of Cu_SG_CAC_Ibu and Cu_SG_CIC was evaluated in Tris-HCl medium (pH 7.4) and compared to the copper release profile of the bare samples; samples were incubated at 37 °C up to 14 days and, at selected time points (1 h, 3 h, 5 h, 8 h, 24 h, 2 d, 3 d, 8 d, 10 d, 14 d) the suspension was centrifuged and the supernatant withdrawn and analyzed by ICP-AES. As shown in Figure 8, a significantly prolonged release profile of up to 14 days was observed for both the Cu_SG_CAC_Ibu and Cu_SG_CIC samples, evidencing that just 30% of the total amount of copper was released after 3 h, compared to the burst release observed for the Cu_SG sample (95% of total amount) followed by a sustained release of copper up to 14 days. The slower copper release rate observed for Cu_SG_CAC_Ibu and Cu_SG_CIC could be ascribed to the long path length, and the slowed-down diffusion of the medium throughout the polymer layers [51], since the polyelectrolyte multi-layer, acting as a barrier, was expected to hinder the contact between the ion and the release medium. The build-up of multi-layers on Cu-substituted MBGs successfully decreased the undesirable initial burst release of Cu^2+^ species typically observed for particles as such. In fact, after 1 h incubation in similar releasing conditions, Cu_SG_CAC_Ibu and Cu_SG_CIC released an amount of copper ions approximately 80% and 70% lower compared to the corresponding bare MBG particles, confirming the ability of the multi-layered coating to prevent the typical burst release effect observed for the bare MBG particles. In addition, at the end of the experiment (14 days), Cu_SG_CAC_Ibu and Cu_SG_CIC particles were still present in the medium and, as observed in Figure 8, the release curve did not reach the plateau. These observations could suggest that the dense polyelectrolyte multi-layer effectively acts as a barrier, limiting the interaction of MBG surface with the medium, thus slow downing the ion-exchange reaction and the overall dissolution processes. For this reason, after 14 days, the system was still capable of releasing copper ions, further confirming that the presence of the multi-layered coating can be exploited to produce multifunctional devices with a sustained release over 14 days.

It is worth highlighting that the release kinetics observed for the Cu_SG_CAC_Ibu and results were slower compared to the Cu_SG_CIC and this behavior can be ascribed to the denser and ticker polymer network obtained after the chitosan/alginate/chitosan deposition, more effectively able to hinder the contact between the particles and the medium. On contrary, the less closely packed of the chitosan/ibuprofen/chitosan coating allowed faster medium diffusion and thus the copper release.

### 3.4. Ibuprofen Release from Cu_SG_CAC_Ibu and Cu_SG_CIC

**Figure 9:**
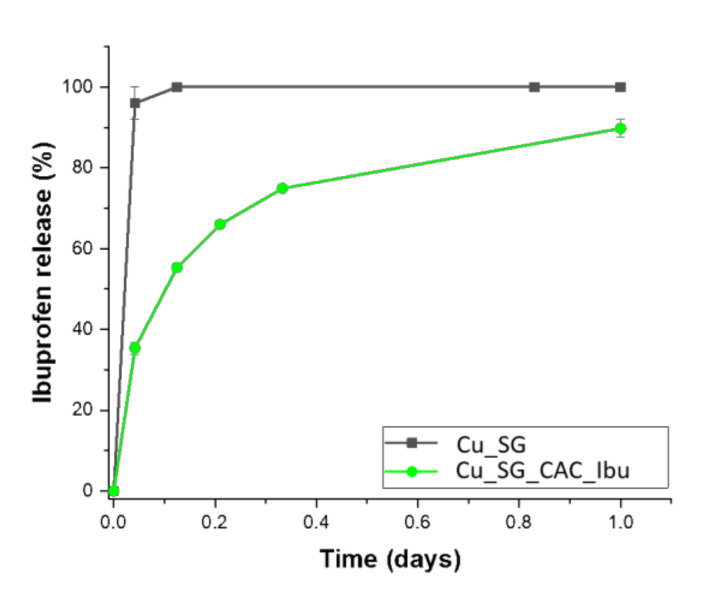
Ibuprofen release profiles of Cu_SG_CAC_Ibu compared with bare samples.

Ibuprofen release test was performed in Tris-HCl up to 24 h. Figure 9 showed the release profile of ibuprofen from Cu_SG_CAC_Ibu powders compared to the corresponding bare samples. An evidently sustained release profile of loaded drug up to 1 day of soaking in the release medium was observed, thus confirming the ability of the multi-layered coating to modulate the burst release typically reported for mesoporous carriers. In fact, compared to the release profile of the corresponding bare samples in which the total amount of loaded ibuprofen was released in the first hour, only 38% of the total amount of loaded drug was released after one hour of soaking from Cu_SG_CAC_Ibu, suggesting a significant slowdown in the release rate.

The modulation in release rate could be ascribed to the electrostatic interactions between the drug and the polyelectrolyte multi-layers. In fact, the strong ionic interactions between the amine groups of the chitosan and the carboxyl groups of the ibuprofen could potentially hinder the diffusion pathways of ibuprofen among the layers, thus delaying the contact between the drug and the release medium, as already reported for the copper release. In addition, the presence of the polyelectrolyte multi-layer increases the diffusional path, thereby causing the tendency for the delayed release of ibuprofen.

**Figure 10:**
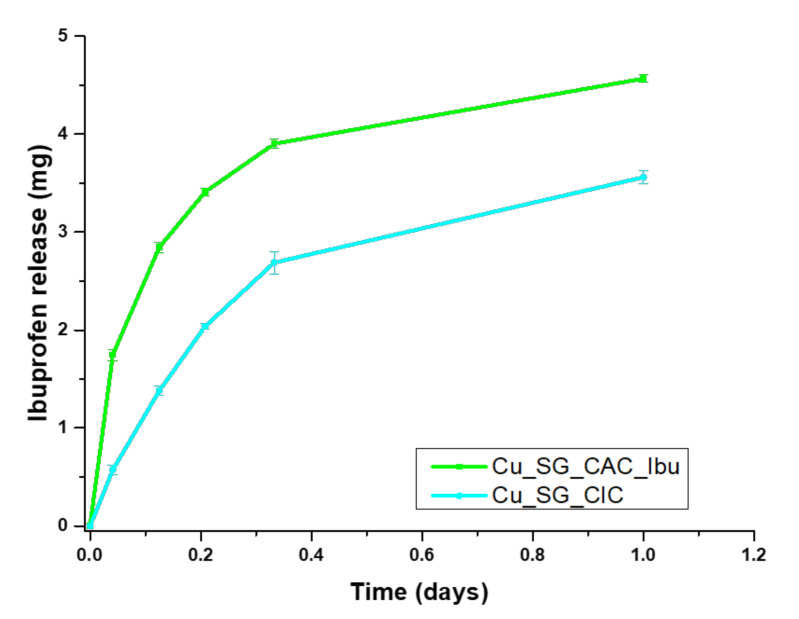
Ibuprofen release profiles of Cu_SG_CIC compared with Cu_SG_CAC_Ibu samples.

The release profile of ibuprofen from Cu_SG_CIC was similarly evaluated in Tris-HCl. Figure 10 showed the release profile of ibuprofen from Cu_SG_CIC in terms of milligrams of released drug overtime, and not as a percentage of ibuprofen released since the amount of drug effectively incorporated could not be clearly evaluated by the TGA, probably due to the decomposition of chitosan, which occurred in the same range as ibuprofen. As shown in Figure 10, different drug release curves were obtained depending on the method used to incorporate ibuprofen. In fact, a more sustained release profile can be observed for the Cu_SG_CIC system, compared with the Cu_SG_CAC_Ibu. This behavior could probably be ascribed to the direct interactions established between the carboxylate groups of ibuprofen and the protonated amines of chitosan during the assembly.

In addition, since the drug release was a mainly diffusion-controlled process, as confirmed by many previous studies [23,52,53], the presence of a multi-layered coating can hinder the contact between the drug and the medium, thus delaying the diffusion of the ibuprofen into the release medium. It was worth noticing that, also, in this case, the release kinetics of the CIC samples was slower compared to the bare samples, demonstrating that the presence of hydrogen bonding between chitosan and ibuprofen and the presence of the multi-layered coating, which act as a barrier between the drug and the medium could effectively modulate the ibuprofen release rate.

Since the main goal of this work was the development of an advanced, versatile drug/ion release platform in which the release was modulated by the LbL deposition, in order to validate the release profile in biological conditions and the synergy of the two therapeutic agents able to simultaneously induce tissue healing and an anti-inflammatory effect, in vitro and in vivo experiments need to be properly planned. However, to ensure its effectiveness, the released concentration of ibuprofen needs to fall within the proper therapeutic window. Shah et al. reported that ibuprofen exerted its anti-inflammatory effects when the concentration was >50 µg/mL [54]. Both the designed systems, in the experimental conditions selected for the release test in vitro, perfectly matched this concentration from the first hour of release. However, depending on the final application, a fine optimization of the ibuprofen deposited as well as of the amount of ibuprofen loaded through the incipient wetness method could allow the design of systems releasing an efficacious and cytocompatibility amount of ibuprofen over time. In fact, according to Cantòn et al., ibuprofen exerts its anti-inflammatory effects with no cytotoxicity within the concentration range 0.1–1 mM [55]. In addition, two different applications and corresponding dosages of ibuprofen can be distinguished [56,57]: within 0.1 and 0.14 mM concentrations, ibuprofen was reported to exert anti-inflammatory, antipyretic, and analgesic effects, meanwhile higher dosages within the range 0.2–0.3 mM were required to treat chronic diseases such as rheumatoid arthritis and osteoarthritis. The potentiality and versatility of the developed drug/ion release platforms rely on the modulation of both the timing and the dosage of the ibuprofen release in order to target the requirements of each specific application, thus allowing to obtain the proper therapeutic effect and avoiding cytotoxicity.

## 4. Conclusions

In this work, innovative multifunctional drug delivery systems for the delivery of both copper ions (exerting pro-angiogenic and anti-bacterial effects) and an anti-inflammatory drug (ibuprofen) were developed by exploiting the layer-by-layer deposition technique. To this purpose, the surface of Cu-substituted MBG (2% mol) nanoparticles was modified by using two different approaches. On the one hand, the polyelectrolytes chitosan and alginate have been alternatively deposited on the MBG surface, and ibuprofen was loaded by using the incipient wetness technique. On the other hand, the alginate layer was replaced by ibuprofen molecules, loaded as polyelectrolyte layer. Irrespective of the adopted procedures, the bioactive behavior of the particles was maintained, preserving an essential feature for promoting bone regeneration. Both the investigated procedures allowed to modulate the release kinetic of both copper and ibuprofen, allowing a sustained ion/drug co-release. In fact, a prolonged copper release profile up to 14 days was observed, with a strong reduction of the burst release in the first hours of soaking compared to the bare analog sample. The release modulation can be associated to the long path length and the amount of time needed for the ions to diffuse into the polymer layers before reaching the medium. In addition, the release of copper from the CAC-coated samples resulted slower compared to the CIC-coated samples, probably due to the denser polymer network obtained after the chitosan/alginate/chitosan deposition that hinders the contact between the particles and the medium. On the contrary, the more open network of the chitosan/ibuprofen/chitosan coating is expected to allow more easily the medium diffusion and thus the copper release. Concerning the ibuprofen release, sustained release profile up to 1 day of soaking was reached for both the systems, evidencing the role of the LbL coating in strongly reducing the burst release observed for the bare samples, most likely due to the occurrence of ionic interactions between the protonated amine groups of the chitosan and the carboxylate groups of ibuprofen.

Overall, the obtained results proved layer-by-layer deposition as a promising technique to design a drug delivery system that modulates the drug and therapeutic ion release from MBGs. In addition, by tailoring the number of the deposited layers or the amount of the loaded drug, versatile drug delivery systems can be developed as a smart platform for soft and hard tissue healing applications. With this perspective, a comprehensive biological validation of the developed LbL coated nanocarriers, including cytotoxicity assessment, the evaluation of the ion/drug co-release under biological conditions and the exerted synergistic therapeutic effect (simultaneously pro-angiogenic, anti-inflammatory, and anti-bacterial) will be the focus of further research investigation by the authors.

## Figures and Tables

**Table 1:** The specific surface area of Cu_SG, Cu_SG_CAC_Ibu and Cu_SG_CIC.

Sample	Specific Surface Area (m^2^/g)
Cu_SG	685
Cu_SG_CAC_Ibu	125
Cu_SG_CIC	276

**Table 2:** Weight loss percentages associated with each deposited layer and ibuprofen loading.

Cu_SG_CAC_Ibu		Cu_SG_CIC	
Layer	Weight Loss (%)	Layer	Weight Loss (%)
chitosan	6.6	chitosan	6.6
alginate	7.5	ibuprofen	0.2
chitosan	5.8	chitosan	6.1
loaded ibuprofen	10.5		

**Table 3:** Potential (mV) values of Cu_SG_CAC_Ibu, and Cu_SG_CIC after each layer deposition.

Cu_SG_CAC_Ibu		Cu_SG_CIC	
Cu_SG	−13 ± 0.6	Cu_SG	−13 ± 0.6
Chitosan	50 ± 3.8	Chitosan	41 ± 3.8
Alginate	−29 ± 3.2	Ibuprofen	−21 ± 3.4
Chitosan	21 ± 2.2	Chitosan	27 ± 5.0

## Supplementary Materials

The following are available online at https://www.mdpi.com/article/10.3390/pharmaceutics13111952/s1, Figure S1: (A) TEM image and (B) FE-SEM image of un-coated Cu_SG.
