# Supplementary material for: Polyelectrolyte-Coated Mesoporous Bioactive Glasses via Layer-by-Layer Deposition for Sustained Co-Delivery of Therapeutic Ions and Drugs
Source: Pharmaceutics. 2021 Nov 17;13(11):1952. doi: 10.3390/pharmaceutics13111952 (PMC8625996; doi:10.3390/pharmaceutics13111952)
Supplement: Supplementary file 1 [file pharmaceutics-13-01952-s001.zip › pharmaceutics-1426442-supplementary.pdf]

pharmaceutics

MDPI

Supplementary Materials

Polyelectrolyte-Coated Mesoporous Bioactive Glasses via Layer-by-Layer Deposition for Sustained Co-Delivery of Therapeutic Ions and Drugs

Carlotta Pontremoli [1,2], Mattia Pagani [1], Lorenza Maddalena [3], Federico Carosio [3], Chiara Vitale-Brovarone [1] and Sonia Fiorilli [1,*]

[1] Department of Applied Science and Technology, Politecnico di Torino, Corso Duca degli Abruzzi 24, 10129 Torino, Italy; carlotta.pontremoli@unito.it (C.P.); mattia.pagani@polito.it (M.P.); chiara.vitale@polito.it (C.V.-B.)
[2] Department of Chemistry, NIS Interdepartmental and INSTM Reference Centre, University of Torino, via Giuria 7, 10125 Torino, Italy
[3] Department of Applied Science and Technology, Politecnico di Torino, Alessandria Campus, Viale Teresa Michel 5, 15121 Alessandria, Italy; lorenza.maddalena@polito.it (L.M.); federico.carosio@polito.it (F.C.)
* Correspondence: sonia.fiorilli@polito.it

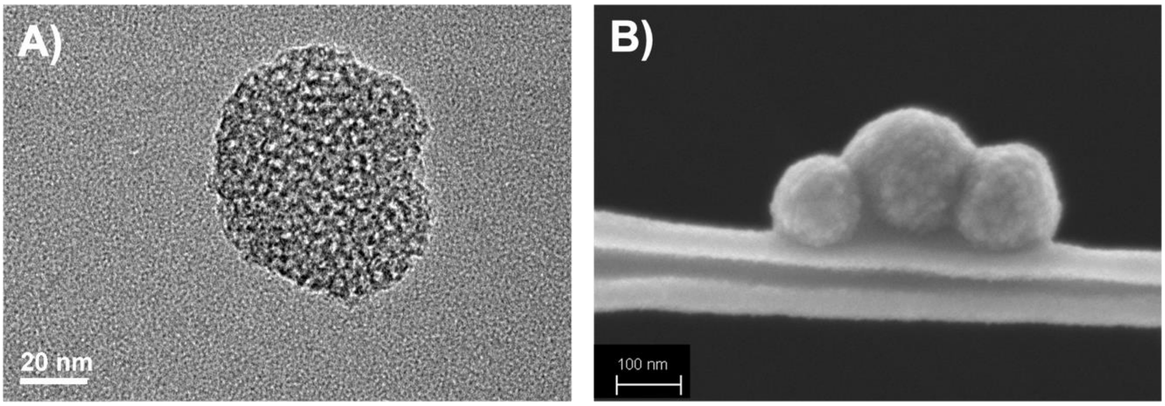

Figure S1. A) TEM image and **B**) FE-SEM image of un-coated Cu_SG.
